# Interleukin-6 knockout reverses macrophage differentiation imbalance and alleviates cardiac dysfunction in aging mice

**DOI:** 10.18632/aging.103749

**Published:** 2020-10-25

**Authors:** Yuan Wang, Shan Zhu, Wen Wei, Yi Tu, Chuang Chen, Junlong Song, Juanjuan Li, Changhua Wang, Zhiliang Xu, Shengrong Sun

**Affiliations:** 1 Department of Thyroid Breast Surgery, Renmin Hospital of Wuhan University, Wuhan 430060, China; 2 Basic Medical School of Wuhan University, Wuhan 430060, China

**Keywords:** aging, interleukin-6, cardiac dysfunction, macrophages differentiation, cardiomyocyte apoptosis

## Abstract

Several interleukins (ILs) have been shown to be involved in aging, but the effects of IL-6 on aging-related cardiac dysfunction remain unknown. In this study, the expression and sources of cardiac IL-6 in aging hearts were investigated for the first time. The results showed that cardiac IL-6 expression in mice gradually increased with age, and the expression at 16 months, 20 months and 25 months was higher than that at 3 months. In addition, cardiac macrophages (Møs) were shown to be the main sources of IL-6 in aging mice. IL-6 knockout (KO) significantly alleviated cardiac dysfunction, increased M2 macrophage (Mø2) differentiation, reduced M1 macrophage (Mø1) differentiation and protected against cardiomyocyte apoptosis in aging mice. IL-6 KO also reversed the stimulatory effect of doxorubicin (DOX) treatment on Mø1s and the inhibitory effect of DOX treatment on Mø2s *in vitro*. Furthermore, the mRNA expression of both aging markers and apoptosis-related markers was markedly inhibited by IL-6 KO. Our results suggest that aging can be significantly reversed by IL-6 KO and that the mechanisms of this effect are related to alleviation of Mø1/Mø2 imbalance and protection against apoptosis in cardiomyocytes.

## INTRODUCTION

Aging, an inevitable physiological process that occurs in all animals, including humans, can cause organ structural changes, chronic fibrosis, and decreased function [1, 2]. Cardiac aging, which is regulated by the inflammatory response, is one of the most important determinants of cardiac dysfunction, and reversal of cardiac aging has been demonstrated to significantly ameliorate cardiac dysfunction and prolong survival [3]. The mechanisms by which aging mediates cardiac dysfunction are complicated and involve various pathological effects, including reactive oxygen species accumulation, mitochondrial dysfunction, autophagy, and especially the inflammatory response [4].

Interleukins (ILs) are a kind of cytokine that can regulate a variety of physiological effects, several of which have been found by both animal studies and clinical experiments to be involved in aging. For example, elevated circulating IL-1β and IL-6 levels have been observed in aged mice and have been shown to be closely associated with neuronal damage during ischemia [5]. In addition, the expression of IL-7 has been found to be decreased in the aging population; one study found that a group exhibiting low IL-7 expression showed a lower 10-year survival rate than a group exhibiting high IL-7 expression, possibly due to increased activation of immune responses [6]. In mice, up-regulation of IL-10 expression has been reported to prevent skeletal muscle aging by attenuating inflammatory responses and reducing insulin resistance [7]. The multiphasic IL-12 gene has been demonstrated to affect cognitive impairment in aging men [8]. In a 23-month-old mouse model, IL-4 and IL-13 injection into the hippocampus has been found to significantly affect the differentiation of macrophages (Møs), inhibit the expression of M1 macrophages (Mø1s), and promote the expression of M2 macrophages (Mø2s) [9]. Furthermore, plasma IL-15 levels have been found to be decreased in elderly individuals, and treatment with a low dose of IL-15 can promote wound healing in aged mice [10, 11]. In older mice undergoing surgery, IL-17 has been shown to significantly ameliorate cognitive impairment [12]. Finally, a recent study has revealed that knockout (KO) of IL-12p35, a common subunit of IL-12 and IL-35, aggravates both mitochondrial dysfunction and cardiac dysfunction in 25-month-old mice [13].

IL-6, which belongs to the IL-6 superfamily, is a multifunctional cytokine that is secreted by both immune and non-immune cells, especially Møs [14–16]. IL-6 can bind the IL-6 receptor (IL-6R) and gp130, activate the Janus Kinase (JAK)-Signal Transducer and Activator of Transcription 3 (STAT3) signaling pathway, regulate inflammatory responses, and participate in the occurrence and progression of various cardiovascular diseases [14–16]. In a previous study, KO of IL-6 has been observed to reverse angiotensin II (Ang II)-induced hypertension by down-regulating JAK2-STAT3 phosphorylation [17]. In contrast, overexpression of IL-6 significantly increases the expression of tumor necrosis factor α (TNF-α) and aggravates myocardial injury in viral myocarditis mice [18]. In Ang II-infused mice, IL-6 significantly increases cardiac fibrosis and aggravates cardiac hypertrophy [19]. Extensive studies have confirmed that IL-6 can promote the differentiation of a variety of immune cells and then amplify inflammation and regulate ventricular remodeling in myocardial infarction [20]. In addition, agents that inhibit IL-6 activity, such as the antibody tocilizumab, have been used for clinical disease treatment and have benefitted more than one million patients. However, the role of IL-6 in cardiac aging remains unknown. In the present study, IL-6 KO mice were used to determine the roles of IL-6 in aging-related cardiac remodeling and to explore the possible related mechanisms.

## RESULTS

### Aging increases IL-6 expression in cardiac Møs in mice

**Figure 1:**
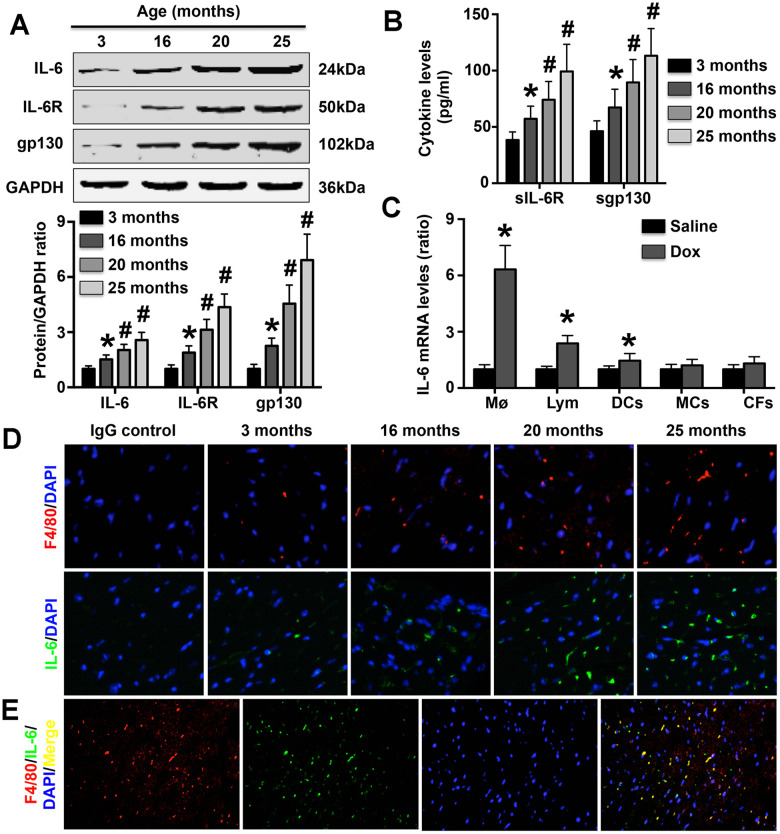
**Effect of aging on cardiac IL-6 expression.** (**A**) The expression of IL-6 in mice at 3 months, 15 months, 20 months, and 25 months of age was measured by Western blotting. N=5 in each group. * p<0.05 vs. the 3-month group. ^#^ p<0.05 vs. the previous group. (**B**) Plasma sIL-6R and sgp130 levels were detected in each group. N=5 in each group. * p<0.05 vs. the 3-month group. ^#^ p<0.05 vs. the previous group. (**C**) Effects of DOX treatment on IL-6 mRNA expression in WT Møs, lymphocytes, DCs, MCs, and CFs. N=5 in each group. * p<0.05 vs. the group. (**D**) The cardiac expression of both IL-6 and Mø markers in mice of different ages was detected in 25-month-old mice by immunofluorescence staining. N=5 in each group. (**E**) Double immunofluorescence staining for anti-F4/80 and anti-IL-6. N=5 in each group.

The results showed that both cardiac IL-6 expression and Mø marker expression in mice gradually increased during aging (as measured at 3 months, 16 months, 20 months and 25 months of age; Figure 1A, 1D). In addition, both soluble and cardiac IL-6R and gp130 expression exhibited a trend similar to that of IL-6 expression (Figure 1A, 1B). Furthermore, IL-6 mRNA expression was significantly increased in Møs and slightly increased in lymphocytes and DCs following DOX treatment but was not altered in MCs or CFs (Figure 1C). Double staining with anti-IL-6 and anti-68 antibodies showed that cardiac Møs were the sources of IL-6 in aging mice (Figure 1E).

### IL-6 deficiency alleviates aging-related cardiac dysfunction in mice

**Figure 2:**
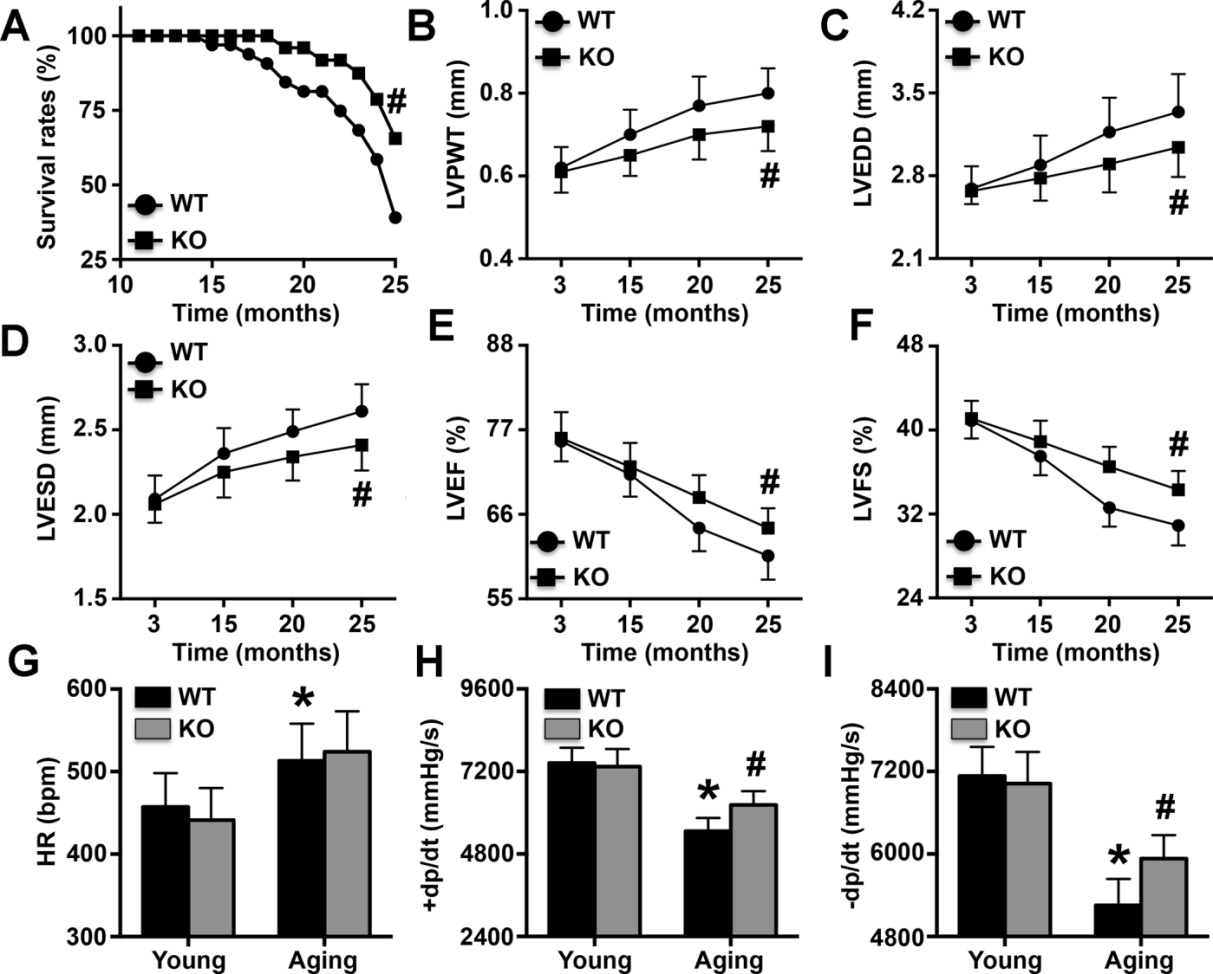
**Effects of IL-6 KO on cardiac dysfunction.** (**A**) Survival rates of WT mice and IL-6 KO mice. (**B**–**F**) LVPWT, LVEDD, LVESD, LVEF, and LVFS were determined at different time points by ultrasonic cardiography. (**G**–**I**) The HRs of young mice and aging mice were measured. N=10-16 in each group. * p<0.05 vs. the young WT group; ^#^ p<0.05 vs. the aging WT group.

At the end of the 25^th^ month, the survival rates of both WT mice and IL-6 KO mice were significantly decreased, with WT mice showing more pronounced decreases in survival (Figure 2A). In addition, LVPWT, LVEDD, and LVESD gradually increased with the aging process, and the increases were more pronounced in WT mice than in IL-6 KO mice (Figure 2B–2D). In contrast, LVEF and LVFS gradually decreased with age, but the changes were again more pronounced in WT mice than in IL-6 KO mice (Figure 2E, 2F). Although the aged mice exhibited higher HRs than the young mice, IL-6 KO did not affect HR (Figure 2G). Furthermore, the aging-induced declines in +dp/dt and +dp/dt were markedly reversed by IL-6 KO (Figure 2H, 2I).

### IL-6 KO relieves cardiac aging dysfunction and reduces aging-related mRNA expression in mice

**Figure 3:**
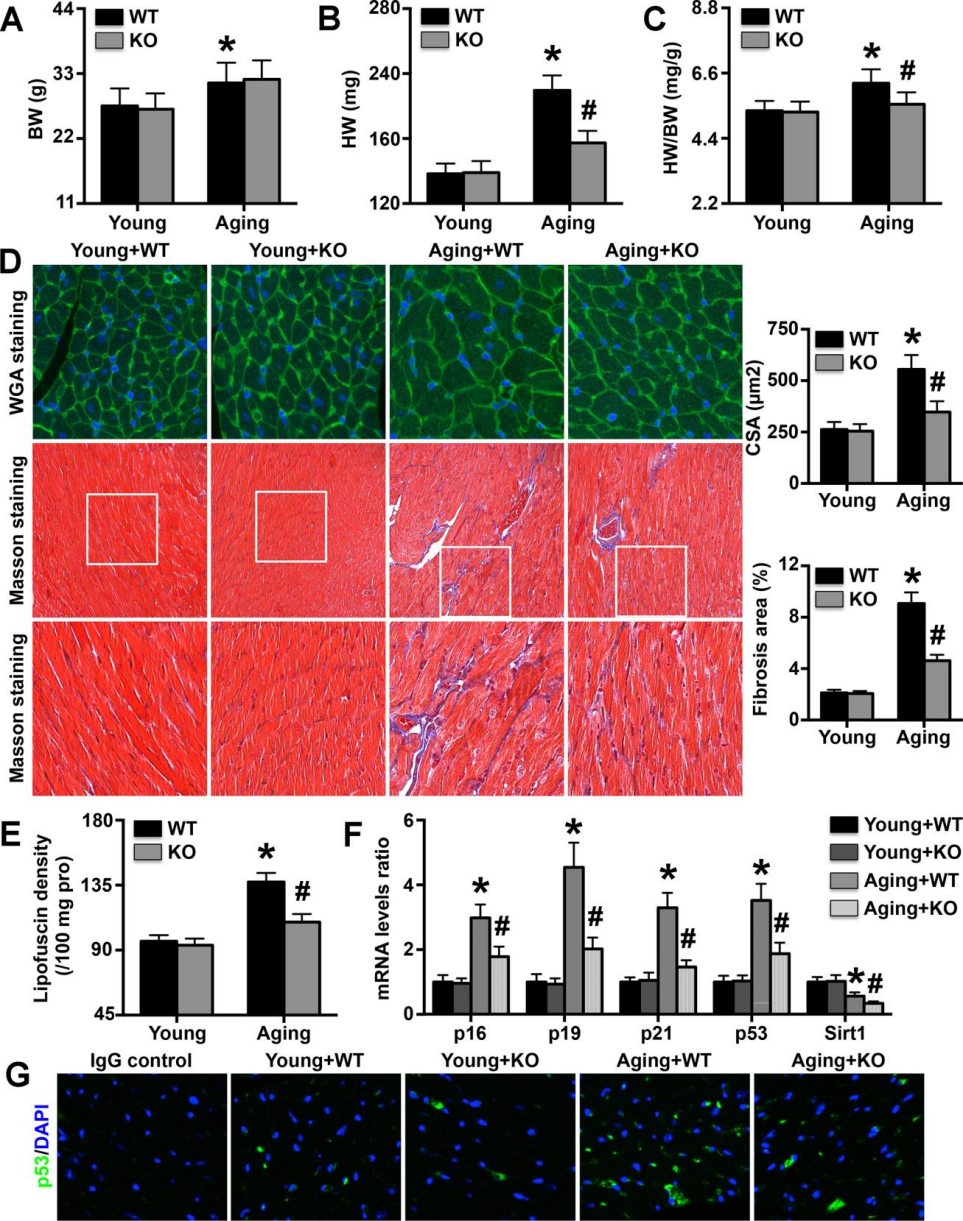
**Effects of IL-6 KO on cardiac remodeling and aging-related protein levels.** (**A**–**C**) Body weights (BWs), heart weights (HWs), and HW/BW ratios in the four groups. (**D**) MC CSA and cardiac fibrosis area in each group. (**E**) Lipofuscin density in each group. (**F**) p16, p19, p21, p53, and Sirt1 mRNA expression levels were analyzed by RT-PCR. (**G**) Cardiac p53 protein expression was detected by immunofluorescence staining. N=5-8 in each group. * p<0.05 vs. the young WT group; ^#^ p<0.05 vs. the aging WT group.

Aging mice exhibited higher body weights than young mice, and IL-6 KO did not affect body weight in either young or aging mice (Figure 3A). However, IL-6 KO attenuated the aging-induced increases in heart weight and the heart weight/body weight ratio (Figure 3B, 3C). The MC CSAs and cardiac fibrosis areas followed trends similar to those of heart weight and the heart weight/body weight ratio (Figure 3D). The cardiac expression of lipofuscin was also elevated in aging mice and was decreased by IL-6 KO (Figure 3E). The expression levels of the aging markers p16, p29, p21 and p53 were increased in aging mice, but the increases were reversed by IL-6 KO; Sirt1 exhibited the opposite trends (Figure 3F). The immunofluorescence staining results showed that cardiac p53 protein expression followed the same trend as p53 mRNA expression (Figure 3G).

### IL-6 deletion reverses Mø1/Mø2 imbalance in aging mice

**Figure 4:**
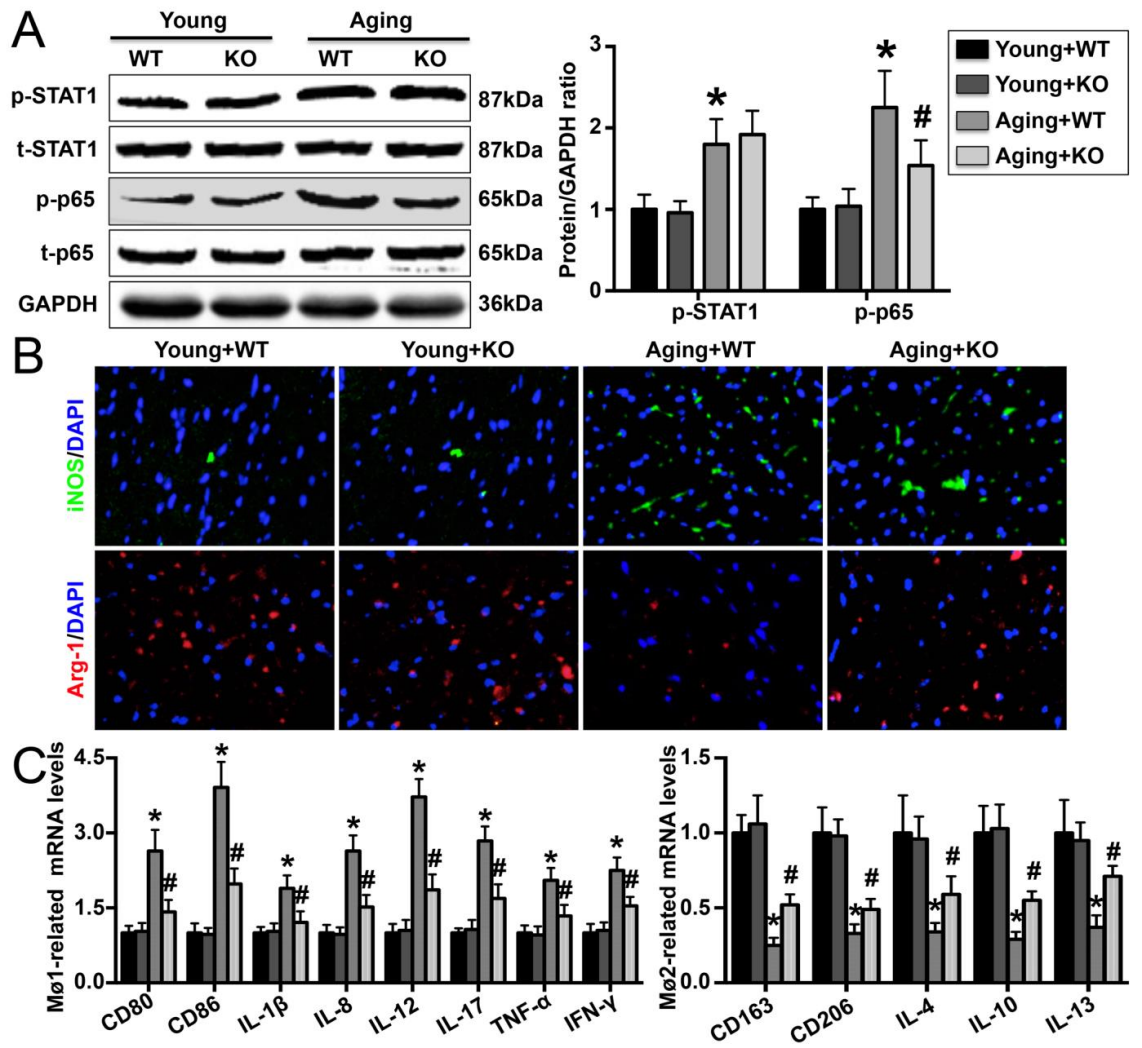
**Effect of IL-6 KO on cardiac Mø differentiation.** (**A**) STAT1 and p65 phosphorylation was detected by Western blotting. (**B**) Cardiac iNOS and Arg-1 levels were measured by immunofluorescence staining. (**C**) mRNA expression of Mø1- and Mø2-related markers in each group. N=5-8 in each group. * p<0.05 vs. the young WT group; ^#^ p<0.05 vs. the aging WT group.

The phosphorylation levels of both STAT1 and p65 were higher in aging mice than in young mice, and IL-6 KO decreased p65 phosphorylation but had no effect on STAT1 phosphorylation (Figure 4A). In addition, IL-6 KO increased cardiac iNOS expression but decreased Arg-1 expression (Figure 4B). Aging IL-6 KO mice showed reduced Mø1-related mRNA (CD80, CD86, IL-1β, IL-8, IL-12, IL-17, TNF-α, and IFN-γ) expression and elevated Mø2-related mRNA (CD163, CD206, IL-4, IL-10 and IL-13) expression (Figure 4C).

### IL-6 KO protects against MC apoptosis in mice

**Figure 5:**
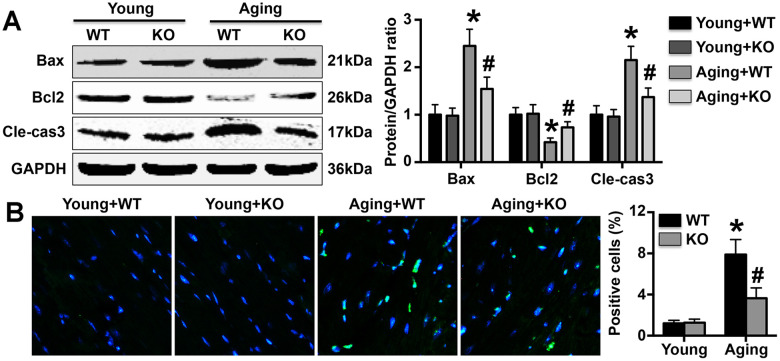
**Effects on MC apoptosis.** (**A**) The expression of apoptosis-related proteins was detected in hearts. (**B**) TUNEL staining was performed to mark apoptotic cells, and the number of TUNEL-positive cells in each group was determined. N=5-8 in each group. * p<0.05 vs. the young WT group; ^#^ p<0.05 vs. the aging WT group.

IL-6 KO reduced Bax and cleaved-caspase3 expression and increased Bcl2 expression in aging mice but did not affect the expression of these apoptosis-associated proteins in young mice (Figure 5A). TUNEL staining revealed that more TUNEL-positive MCs were present in aging mice than in young mice, but the difference was attenuated by IL-6 KO (Figure 5B).

### IL-6 deficiency reduces cardiomyocyte apoptosis associated with the differentiation of Møs *in vitro*

**Figure 6:**
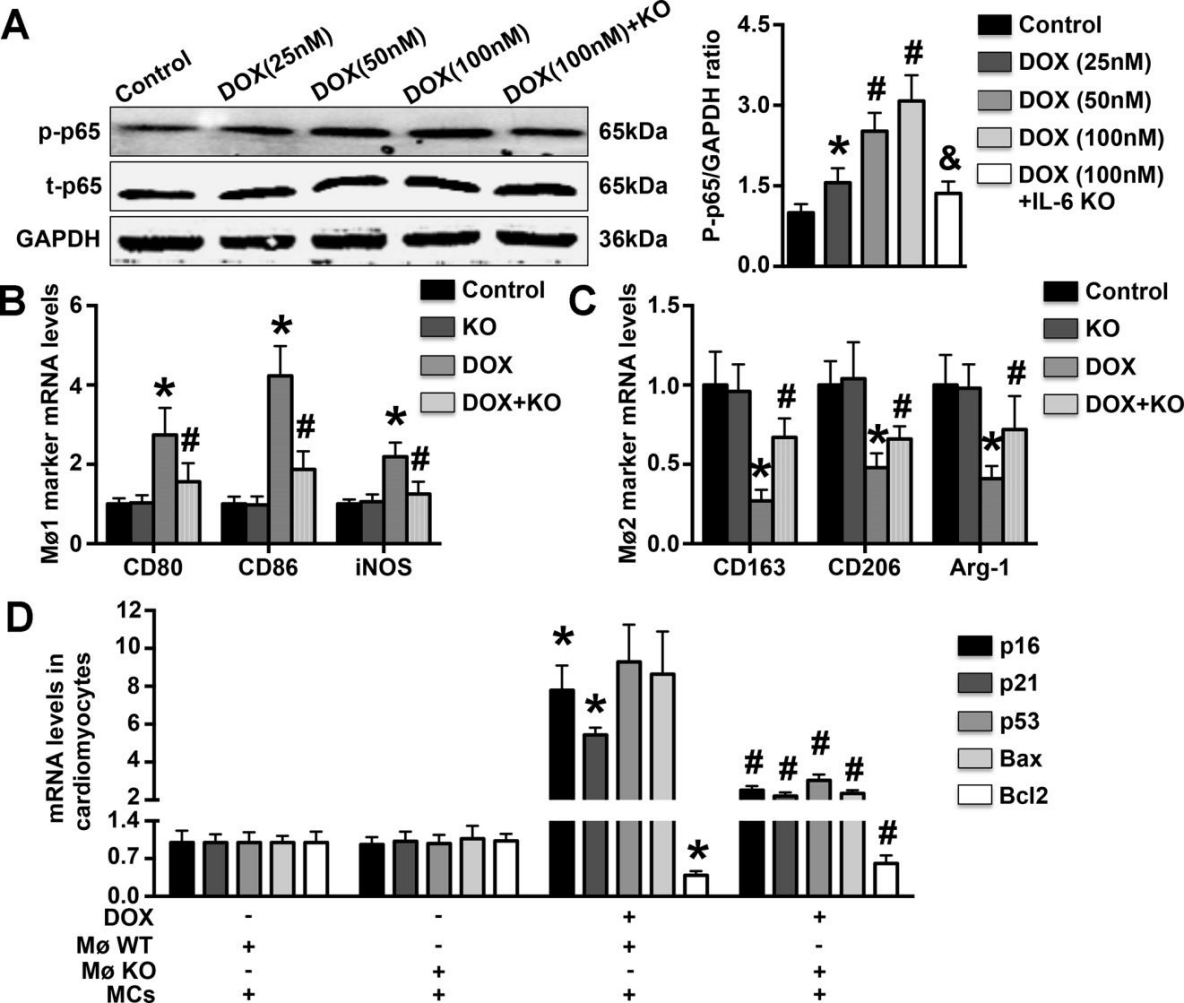
**Effects of IL-6 KO on Mø differentiation and MC apoptosis *in vitro*.** (**A**). The levels of p65 phosphorylation in DOX-treated WT Møs and IL-6 KO Møs were measured. (**B**, **C**). The mRNA expression of Mø1- and Mø2-related markers was measured in DOX-treated Møs. (**D**). The effects of DOX-induced Mø differentiation on MC apoptosis and MC aging were determined by RT-PCR. N=5 in each group. ^*^ p<0.05 vs. the young WT group; ^**#**^ p<0.05 vs. the aging WT group.

DOX treatment dose-dependently increased p65 phosphorylation in WT Møs, and this effect was significantly reversed by IL-6 KO (Figure 6A). In addition, DOX treatment increased CD80, CD86, and iNOS mRNA expression but decreased CD163, CD206, and Arg-1 mRNA expression, and the regulatory effects of DOX on these Mø markers were reversed by IL-6 KO (Figure 6B, 6C). Furthermore, treatment with the supernatant of DOX-treated WT Mø culture medium significantly increased p16, p21, p53 and Bax mRNA expression and decreased Bcl2 mRNA expression in MCs, while treatment with the supernatant of DOX-treated IL-6 Mø culture medium reduced p16, p21, p53, and Bax mRNA expression but increased Bcl2 mRNA expression (Figure 6D).

## DISCUSSION

In the present study, we examined the expression and sources of IL-6 in the aging heart for the first time. We found that IL-6 was secreted by cardiac Møs and that IL-6 expression gradually increased with the aging process. In addition, more Mø2s and higher levels of Mø2-related markers and inflammatory factors, as well as fewer Mø1s and lower levels of Mø1-related markers and inflammatory factors, were observed in IL-6-deficient aging mice than in WT aging mice. IL-6 deletion also alleviated aging-induced cardiac remodeling and dysfunction. Furthermore, IL-6 KO inhibited Mø1 differentiation and promoted Mø2 differentiation under DOX treatment *in vitro*. Treatment of MCs with medium from DOX-treated IL-6 KO Møs also alleviated the increases in the mRNA expression of aging markers and apoptosis markers.

IL-6, a pro-inflammatory cytokine, has been observed to play roles in a variety of human and mouse cardiovascular diseases. Plasma IL-6 concentrations have been measured in both hypertensive patients and an Ang II-induced mouse hypertension model [17, 21]. Significantly increased IL-6 mRNA expression has also been found in the fibrotic hearts of mice subjected to Ang II via infusion [19, 22]. In addition, IL-6 expression has been reported to be elevated in Coxsackievirus B3-induced mouse viral myocarditis and in acute aortic dissection patients [18, 23]. In the present study, we found that cardiac IL-6, IL-6R, and gp130 expression levels in mice gradually increased over time. Since sIL-6R and sgp130 are critical for initiating the biological effects of IL-6 [24, 25], we also detected their expression, and the results were similar to those for IL-6, consistent with previous findings related to human aging [26]. These findings suggest that IL-6 is involved in cardiac aging. In previous studies, DOX treatment has often been used to simulate aging *in vitro* [3, 27]. Therefore, some immune and non-immune cell types were treated with DOX in this study to determine the sources of IL-6 in the aging heart. After treatment with DOX, IL-6 mRNA expression was most significantly increased in Møs. These results suggest that cardiac IL-6 is derived mainly from Møs, as confirmed by immunofluorescence staining. Møs are important immune cells, and activated Møs can regulate the inflammatory response and participate in the pathological processes of a variety of diseases. Given previous observations of imbalance between anti-inflammatory and pro-inflammatory cytokines in the aging heart [28, 29], our results may indicate that IL-6 participates in cardiac aging by regulating the inflammatory response.

Cardiac aging can cause a series of characteristic changes, including structural remodeling of the heart, declines in systolic and diastolic function, and changes in the expression of aging markers [13, 30]. The expression of the pro-senescence markers p16, p19, p21, and p53 is increased during aging, while that of the anti-senescence marker Sirt1 is decreased [13]. In addition, lipofuscin expression is significantly increased in aging hearts and has been found to be related to the degree of aging [30]. To detect the effects of IL-6 KO on cardiac aging, we investigated changes in cardiac structure, cardiac function and senescence marker mRNA expression. The results showed that IL-6 KO significantly reversed cardiac hypertrophy, alleviated cardiac dysfunction, decreased pro-senescence marker mRNA expression and increased anti-senescence marker expression. These results suggest that IL-6 deficiency alleviates aging-related cardiac remodeling and that the protective effects may be associated with aging delay. The results also suggest that dynamic changes in the expression of anti-aging and pro-aging genes play important roles in senescence and senescence-related cardiac dysfunction. Down-regulation of pro-aging protein expression or up-regulation of anti-aging protein expression can ameliorate aging-related cardiac dysfunction.

In modern medicine, activated Møs are classified into two types: pro-inflammatory Mø1s, which can secrete a variety of pro-inflammatory cytokines and mediate tissue damage, and anti-inflammatory Mø2s, which play anti-inflammatory roles and mainly mediate tissue repair [31]. Mø populations are not constant in the context of cardiovascular disease but rather change dynamically [32]. Dynamic changes in Mø populations have also been found in the aging heart; however, unlike the changes in Mø populations that occur during cardiovascular disease, gradual increases in Mø1 populations and decreases in Mø2 populations occur in the context of aging, indicating that Mø1/Mø2 imbalance gradually increases during the aging process [33]. Numerous studies have confirmed that IL-6/IL-6R can bind to gp130 and promote the differentiation of Møs into Mø1s. Inhibition of the IL-6 pathway or blockade of downstream gp130 signaling reduces Mø1 differentiation and increases Mø2 differentiation [25, 34]. These findings suggest that IL-6 is an important cytokine that regulates Mø differentiation. In our study, we found that cardiac p65 phosphorylation, which is critical for Mø1 differentiation, was reduced in aging IL-6-deficient mice. In addition, the mRNA expression of iNOS and Mø1-related cytokines was decreased by IL-6 KO, while that of Arg-1 and Mø2-related cytokines was increased. These results suggest that deletion of IL-6 significantly reduces the Mø1/Mø2 imbalance that occurs during cardiac senescence and that these effects play protective roles against aging-related cardiac dysfunction.

Excessive apoptosis of cardiomyocytes has been observed in the aging heart, and attenuation of such apoptosis has been shown to be beneficial and to protect against cardiac dysfunction [13, 30]. This finding suggests that excessive cardiomyocyte apoptosis is an essential process associated with senescence-related cardiac dysfunction. We measured the percentage of apoptotic cells in each group and observed fewer apoptotic cardiomyocytes in aging IL-6 KO mice than in WT aging mice. These results suggest that IL-6 deficiency reduces aging-related cardiomyocyte apoptosis. We also used DOX to simulate aging *in vitro*, and the findings further confirmed our prior results: IL-6 KO significantly reversed the regulatory effects of DOX on Mø differentiation and Mø-related inflammatory marker expression *in vitro*. In addition, the expression of aging markers and apoptotic markers was significantly reduced after treatment with the supernatant of DOX-treated IL-6 KO Mø culture medium. These results may indicate that the significant Mø1/Mø2 imbalance and related aggravation of the inflammatory response that occur in the aging heart are important mechanisms promoting aging and MC apoptosis and that IL-6 deletion can significantly reverse these effects to slow the aging process and protect against cardiac dysfunction. Notably, the expression of both gp130 and IL-6 increased gradually in aging mice, suggesting that the protective effects of IL-6 KO against aging-related cardiomyocyte apoptosis and cardiac dysfunction may be mediated by the gp130 signaling pathway. In the first study to investigate the role of the gp130 pathway in heart failure using a transverse aortic constriction (TAC) mouse model), Hirota et al. found that cardiac-specific gp130 KO increases apoptosis and decreases cardiac function [35]. These results are not consistent with our conclusions, perhaps because different models were used.

Collectively, our results show that IL-6 deficiency in mice significantly alleviates aging-related cardiac remodeling and cardiac dysfunction. Our results also demonstrate that IL-6 deficiency may reduce Mø1/Mø2 imbalance and attenuate the inflammatory response, thus slowing the aging process and ameliorating excessive cardiomyocyte apoptosis. Given the inevitability of the aging process and the detrimental effects of this process on cardiac function, KO of IL-6 and further down-regulation of the inflammatory response may be beneficial for delaying aging-related cardiac damage and cardiac dysfunction.

## MATERIALS AND METHODS

### Animals and animal model

Heterozygous IL-6 KO mice with a C57BL/6J background were purchased from the Institute of Model Zoology of Nanjing University (China), housed in a pathogen-free mouse room at Renmin Hospital of Wuhan University and given a normal diet and water. Heterozygous IL-6 KO mice were mated to obtain homozygous IL-6 KO mice and wild-type (WT) mice, both of which were used in this study. First, WT mice at different ages (3 months, 15 months, 20 months and 25 months) were euthanized for analysis of cardiac IL-6 expression (n=10 for each group). In addition, 3-month-old WT mice (n=23) and IL-6 KO mice (n=31) were fed until they reached 25 months of age, at which point they were defined as aged mice; other young mice (n=10 for both WT mice and IL-6 KO mice) at 3 months of age were used as controls. The body weights and heart weights of all mice were recorded, and cardiac remodeling and cardiac Mø differentiation were detected in each mouse. This research was approved by the Ethics Committee of Wuhan University.

### Analyses of left ventricular cardiac structure and function

Both echocardiography and hemodynamic analysis were used to determine the structure and function of the left ventricle. First, the left ventricular posterior wall thickness (LVPWT), left ventricular end-diastolic diameter (LVEDD), left ventricular end-systolic diameter (LVESD), left ventricular ejection fraction (LVEF) and left ventricular fractional shortening (LVFS) were measured in both WT mice and IL-6 KO mice using a MyLab 30CV ultrasound (Esaote SpA, Genoa) system with a 10-MHz linear array ultrasound transducer at different time points (3 months, 15 months, 20 months and 25 months). At the end of the 25-month experimental period, all aged WT mice and young control mice were anesthetized with 2% isoflurane, and a microtip catheter transducer (Millar, Inc., Houston) was inserted into the left ventricle through the right carotid artery to analyze the heart rate (HR), maximal slope of the systolic pressure increment (+dp/dt max), and diastolic pressure decrement (-dp/dt max) using a Millar Pressure-Volume System (Millar, Inc.).

### Detection of left ventricular protein expression

Both left ventricular tissue and Møs were lysed, and the total protein was obtained. All the protein samples were quantified and adjusted to the same concentration using a BCA protein assay kit (Thermo Fisher Scientific). Then, gel electrophoresis was performed to separate the proteins with different molecular weights, and the separated proteins were transferred to Immobilon-FL PVDF membranes (Millipore). After blocking the membranes with nonfat milk, the expression of IL-6 (GTX110527, 1:1000), IL-6R (GTX53204 1:500), gp130 (ab202850, 1:500), Bax (GTX109683, 1:1000), Bcl2 (GTX100064, 1:1000), cleaved-caspase3 (ab32042, 1:500), p-STAT1 Ser727 (GTX132507, 1:500), t-STAT1 (GTX64344, 1:1000), p-p65 (GTX54672, 1:500), t-p65 (GTX102090, 1:1000), and GAPDH (ab181602, 1:5000) was detected using appropriate antibodies (purchased from GeneTex or Abcam). After the membranes were treated with secondary antibodies, the protein expression was analyzed.

### Detection of soluble IL-6R (sIL-6R) and soluble gp130 (sgp130) levels

Serum samples were obtained via centrifugation of blood samples and then stored at -80°C until further analysis. After the serum samples were thawed at 4°C, the sIL-6R and sgp130 levels in each sample were measured using a Milliplex Map Kit (EMD Millipore) following the manufacturer’s recommendations.

### Analysis of lipofuscin

Separated hearts were rapidly frozen and then homogenized with a 1:20 chloroform:methanol mixture (w:v). After centrifugation at 5000 × *g* for 30 minutes, the serum was collected, and the chloroform-rich layer was isolated. Then, the fluorescence of each sample was detected using a spectrofluorometer. In this study, the lipofuscin concentration is expressed as the fluorescence intensity per 100 mg of left ventricular tissue.

### Histological analysis

After mice were euthanized, their whole hearts were rapidly separated, placed into a 15% KCl solution to induce arrest during diastole, and then fixed in 4% neutral paraformaldehyde for 3 days. The hearts were then embedded, cut into 4- to 5-μm slices and mounted onto slides. The cardiomyocyte cross-sectional area (CSA) was determined via assessment of wheat germ agglutinin (WGA) staining in more than 50 cells in each group. The cardiac fibrosis area in each sample was investigated by Masson staining. A mouse anti-F4/80 antibody (MAB5580, 15 μg/ml, R&D Systems), a mouse anti-IL-6 antibody (GTX110527, 1:200, GeneTex), a mouse anti-inducible nitric oxide synthase (iNOS) antibody (ab213987, 2.5 μg/ml, Abcam), a mouse anti-arginase 1 (Arg-1) antibody (ab239731, 2.5 μg/ml, Abcam), and an anti-p53 antibody (GTX70214, 1:80, GeneTex) were used to mark the expression of cardiac Møs, IL-6, iNOS, Arg-1, and p53, respectively. Immunofluorescence double staining with an anti-F4/80 antibody and an anti-IL-6 antibody was performed to determine whether cardiac Møs were the source of cardiac IL-6. In addition, a terminal deoxynucleotidyl transferase-mediated dUTP nick-end labeling (TUNEL) kit (Sigma) was used to mark apoptotic cardiomyocytes.

### Analysis of mRNA expression in the heart and Møs

Total mRNA was extracted from each left ventricular sample and from cells with TRIzol reagent (Sigma). Then, the total mRNA was reverse transcribed into cDNA using a reverse transcription kit, and PCR amplification was performed using LightCycler 480 SYBR Green Master Mix (Roche) to detect target mRNA expression. The mRNA expression levels of p16, p19, p21, p53, Sirt1, CD80, CD86, IL-1β, IL-8, IL-12, IL-17, TNF-α, IFN-γ, CD163, CD206, IL-4, IL-10, and IL-13 in the left ventricular tissues and IL-6, CD80, CD86, iNOS, CD163, CD206, Arg-1, p16, p19, p21, p53, Bax, and Bcl2 in the cells were detected. All the target gene expression levels were normalized to the expression levels of GAPDH. The sequences of the primers used for RT-qPCR are shown in Table 1.

**Table 1:** Primer sequences used for real-time quantitative RT-PCR.

**Gene**	**Forward primer**	**Reverse primer**
p16	CAGATTCGAACTGCGAGGA	CAGCGGAACACAAAGAGCA
p19	GAGAGGGTTTTCTTGGTGA	AGAAGAGCTGCTACGTGA
p21	ATGTCCAATCCTGGTGATGT	TGCAGCAGGGCAGAGGAAGT
p53	GAGCTCCCTCTGAGCCAGGA	TGGGCCTTCAAAAAACTCCTCA
Sirt1	TATCTATGCTCGCCTTGCGG	CGGGATATATTTCCTTTGCAAACTT
CD80	CCATGTCCAAGGCTCATTCT	TTCCCAGCAATGACAGACAG
CD86	CAACGGAATTAGGAAGAC	CTCTGTATGCAAGTTTCC
CD163	CAGGTGTTATCTGCTCCGAGTTC	CCCCATGTACCATTGTAAAACACTT
CD206	GCAAGGAAGGTTGGCATTTGTA	TCCTTTCAGTCCTTTGCAAGC
iNOS	TGACGCTCGGAACTGTAGCA	CAGTGATGGCCGACCTGAT
Arg-1	TGCTGATGGGAGGAGATGTCT	TTTCTTTCAGGGACAGCCTGTT
IL-1β	GGGCCTCAAAGGAAAGAATC	TACCAGTTGGGGAACTCTGC
IL-4	ACGAGGTCACAGGAGAAGGGA	AGCCCTACAGACGAGCTCACTC
IL-6	AGTTGCCTTCTTGGGACTGA	TCCACGATTTCCCAGAGAAC
IL-8	TTCAGAGACAGCAGAGCACA	AGCACTCCTTGGCAAAACTG
IL-10	ATAACTGCACCCACTTCCCA	GGGCATCACTTCTACCAGGT
IL-12	AGTTTGGCCAGGGTCATTCC	TCTCTGGCCGTCTTCACCAT
IL-13	CGCAAGGCCCCCACTAC	TGGCGAAACAGTTGCTTTGT
IL-17	TCCAGAAGGCCCTCAGACTA	AGCATCTTCTCGACCCTGAA
TNF-α	CCCAGGGACCTCTCTCTAATC	ATGGGCTACAGGCTTGTCACT
IFN-γ	ACTGGCAAAAGGATGGTGAC	TGAGCTCATTGAATGCTTGG
Bax	TTGCTGATGGCAACTTCAAC	GATCAGCTCGGGCACTTTAG
Bcl2	CAGAAGATCATGCCGTCCTT	CTTTCTGCTTTTTATTTCATGAGG
GAPDH	CCTCGTCCCGTAGACAAAATG	CAATCTCCACTTTGCCACTGC

### Cell culture experiment

Mouse lymphocytes, dendritic cells (DCs), myocardial cells (MCs), and cardiac fibroblasts (CFs) were all purchased from ScienCell Research Laboratories (USA). Møs were isolated from both WT mice (WT Møs) and IL-6 KO mice (IL-6 KO Møs) at 9-10 weeks of age as described in a previous study [36, 37]. In brief, the femurs were rapidly harvested after the mice had been euthanized, and both ends of the femurs were sheared off. Then, the cells in the femoral canal were flushed using RPMI 1640 culture medium and were plated into 6-well plates after the red blood cells had been lysed. The isolated cells were treated with 20 ng/ml murine macrophage colony stimulating factor (M-CSF) to promote their differentiation into Møs, generating bone marrow-derived Møs. All cells were cultured in RPMI 1640 medium with 10% FBS (both from Gibco).

WT Møs, lymphocytes, DCs, MCs, and CFs were all treated with doxorubicin (DOX, 100 nM) for 24 hours, and cells treated with saline were used as controls. Total mRNA was collected from each cell sample for IL-6 mRNA analysis.

In addition, WT Møs were treated with saline or different doses of DOX doses (25 nM, 50 nM, and 100 nM), and IL-6 KO Møs were also administered DOX (100 nM) [27]. After 24 hours of treatment, the total protein was obtained for detection of p-p65, t-p65 and GAPDH protein levels, and the total mRNA was collected for analysis of the mRNA expression of Mø-related markers.

Finally, MCs were treated with the supernatant of the above culture medium containing 100 nM DOX. Twelve hours later, the mRNA expression of the aging markers and apoptotic markers p16, p21, p53, Bax, and Bcl2 was investigated.

### Statistical analysis

All the data in the present study are expressed as the means ± standard deviations and were analyzed with GraphPad 7. Differences between 2 groups were assessed by Student's t-test, and differences among more than 2 groups were assessed using one-way ANOVA followed by the Newman-Keuls post hoc test. In addition, survival in the aged mice was analyzed by the standard Kaplan-Meier method with the log-rank test. Differences with a value of p < 0.05 were considered significant.
